# Diet and Diversification in the Evolution of Coral Reef Fishes

**DOI:** 10.1371/journal.pone.0102094

**Published:** 2014-07-16

**Authors:** Fabio L. Lobato, Diego R. Barneche, Alexandre C. Siqueira, Ana M. R. Liedke, Alberto Lindner, Marcio R. Pie, David R. Bellwood, Sergio R. Floeter

**Affiliations:** 1 Departamento de Ecologia e Zoologia, Universidade Federal de Santa Catarina, Florianópolis, SC, Brazil; 2 Department of Biological Sciences, Macquarie University, Sydney, NSW, Australia; 3 Setor de Ciências Biológicas, Universidade Federal do Paraná, Curitiba, PR, Brazil; 4 Australian Research Council Centre of Excellence for Coral Reef Studies, and School of Marine and Tropical Biology, James Cook University, Townsville, QLD, Australia; Leibniz Center for Tropical Marine Ecology, Germany

## Abstract

The disparity in species richness among evolutionary lineages is one of the oldest and most intriguing issues in evolutionary biology. Although geographical factors have been traditionally thought to promote speciation, recent studies have underscored the importance of ecological interactions as one of the main drivers of diversification. Here, we test if differences in species richness of closely related lineages match predictions based on the concept of density-dependent diversification. As radiation progresses, ecological niche-space would become increasingly saturated, resulting in fewer opportunities for speciation. To assess this hypothesis, we tested whether reef fish niche shifts toward usage of low-quality food resources (i.e. relatively low energy/protein per unit mass), such as algae, detritus, sponges and corals are accompanied by rapid net diversification. Using available molecular information, we reconstructed phylogenies of four major reef fish clades (Acanthuroidei, Chaetodontidae, Labridae and Pomacentridae) to estimate the timing of radiations of their subclades. We found that the evolution of species-rich clades was associated with a switch to low quality food in three of the four clades analyzed, which is consistent with a density-dependent model of diversification. We suggest that ecological opportunity may play an important role in understanding the diversification of reef-fish lineages.

## Introduction

Explaining the wide differences in species richness among different evolutionary lineages has been a major goal of evolutionary biology since Darwin and still remains one of the greatest challenges of modern biology [1]. The disparate numbers of extant species among clades results from unequal rates of speciation and extinction through time, and recent methodological advances have helped to elucidate the influence of geographical, environmental and biological factors on diversification (e.g., [2]–[4]). For instance, some methods have focused on testing how diversification rates are affected by niche availability in biological communities [4], [5]. In this sense, the concept of density-dependent diversification, also referred to as ‘diversity-dependent’ diversification [6], [7], suggests that speciation rates are faster when there are abundant resources and few competitors [8]. Thus, as niche space becomes saturated over time, speciation rates decline [8]–[11]. Evidence for a diversity-dependent diversification scenario involves recurrent explosive radiations following mass extinction events, and the subsequent decline in diversification rates through time [6]. This general pattern in the fossil record has also been identified in recent phylogenies (e.g., [12], [13]).

We used molecular phylogenies to test the hypothesis of diversity-dependent diversification in reef fishes, one of the most speciose vertebrate assemblages dating back at least to the early Tertiary (50 Ma) [14]. Reef fishes are remarkable for their use of different micro-habitats and food resources across tropical and temperate seas [15]–[17]. In the trophic context, food resources such as algae and sessile invertebrates (e.g. sponges and cnidarians) are abundant but usually present 1) relatively low nutritional value per unit mass [18]–[21], 2) structural and chemical defenses against grazing [22]–[25], and 3) are less digestible compared to mobile invertebrates (e.g. polychaetes, molluscs, crustaceans, echinoderms) and fishes [26]–[28]. The use of low-quality diets would impose specific morphological, physiological, and behavioral constraints on fishes during their evolution, as a result of the energetic costs of this novel feeding mode [29], [30]. Harmelin-Vivien [16] proposed that the relative stability in the tropics, both between seasons and over geological times, may have shaped the evolutionary processes at molecular, organism and community levels leading to a more efficient use of low-quality food resources among tropical reef fish communities. Harmelin-Vivien hypothesized that increasing species richness in the tropics would have led to high competitive pressures among fishes using high-quality diets [16]. In this case, a shift to a nutrient-poor diet that is underutilized by other species would offer a competitive advantage, leading to the rise of recently derived lineages using relatively low-quality diets. To address this hypothesis, we examined the relationship between the trophic status and rates of diversification of reef fishes.

We focused on four representative clades with good phylogenetic resolution (Chaetodontidae, Labridae, Pomacentridae and Acanthuroidei; see [31]) and examined net speciation rates. Specifically, we tested whether niche shifts to the use of relatively low-quality food resources, such as algae and corals are accompanied by increased net diversification of these lineages. Our results show that both predictions – net speciation rates decrease with time and increase following niche shifts to low-quality diets within lineages – are met in three of the four clades analyzed (in the Labridae the changes were recorded but were not restricted to clades with a low quality diet), suggesting that density-dependent diversification may be a general phenomenon in reef fish evolution.

## Materials and Methods

### Reef fish groups

We focused on three speciose families in the “consensus” (archetypical) reef fish families (*sensu* [31]): Labridae, Pomacentridae and Chaetodontidae; and a suborder containing three other reef fish families, the Acanthuroidei. These clades were chosen because they represent important elements in the reef environments and have recent phylogenetic data available to be explored. The Labridae (wrasses and parrotfishes) is a diverse group (∼575 species) with great disparity in body shape, size, coloration, and habitat preference, being most common in shallow habitats such as coral and rocky reefs [32]. Although most labrid species feed on a variety of hard-shelled invertebrates, some lineages use other types of resources, such as algae, detritus, plankton, coral mucus, ectoparasites, and fish [32], [33].

The Pomacentridae (damselfishes and anemonefishes) is one of the four most speciose reef fish families with approximately 380 species [34]. Despite showing great diversity and abundance, damselfishes exhibit a low degree of trophic diversity when compared to the Labridae [15], [32]. This relatively restricted ecological diversity seems to be associated with limited morphological variation [35], and similar biomechanical character states have converged many times during their radiation, leading to convergence on food habits such as herbivory, omnivory and planktivory [36].

The Chaetodontidae (butterflyfishes and bannerfishes) includes over 120 species with spectacular coloration and considerable ecological and morphological diversity [37], [38]. Found on tropical reefs around the world, they prey on a range of taxa, ranging from obligate or facultative coral feeders to omnivores or sessile invertebrate feeders [38], [39].

The Acanthuroidei is a monophyletic suborder that includes the families Acanthuridae (surgeonfishes), Zanclidae (Moorish idol), and Luvaridae (Louvar) [40]. Common in tropical and subtropical seas worldwide, acanthuroids feed mostly by biting attached prey, although zooplanktivory has also evolved within the group [41]. They present a wide variety of morphological and physiological modifications of the jaws and intestines [42]–[44].

### Taxon sampling and sequence alignment

The taxon sampling and molecular data in this study were obtained from previous phylogenetic studies regarding the four abovementioned groups. DNA sequences were downloaded from Genbank following accession numbers in (1) Tang *et al.* [45] for Acanthuroidei, (2) Fessler & Westneat [46] for Chaetodontidae, (3) Westneat & Alfaro [47] for Labridae and (4) Cooper *et al.* [48] for Pomacentridae. Taxon sampling was composed of: (1) 9 acanthuroids; (2) 71 chaetodontids; (3) 84 labrids; (4) 104 pomacentrids.

The sampled Chaetodontidae and Pomacentridae include all currently recognized genera (Table 1). In the suborder Acanthuroidei, all families and genera are represented in the phylogeny. The missing genera in the labrid phylogeny correspond to 12% of labrid species and are mostly monotypic genera (Table 1). Sequences for outgroup species were also downloaded according to the respective references. Sequences downloaded for each species correspond to fragments of (1) two mitochondrial genes (12s, 16s) for acanthuroids, (2) three mitochondrial (12s, 16s, ND3) and two nuclear (Tmo4C4, RAG2) genes for chaetodontids, (3) two mitochondrial (12s and 16s) and two nuclear (Tmo4C4, RAG2) genes for labrids, and (4) three mitochondrial (12s, 16s, ND3) and three nuclear (RAG1, RAG2, bmp4) genes for pomacentrids.

**Table 1:** Number of sampled and missing species from phylogenetic reconstructions (Fig. 1) according to the total number of species in each subclade.

Tree	Subclades	N° species sampled	N° species missing	Total N° species	% species not represented due to missing genera	% species using LQD
Acanthuroidei	Luvaridae	1	0	1	0	0
	Zanclidae	1	0	1	0	0
	Acanthuridae	7	74	81	0	77
	**Total**	**9**	**74**	**83**	**0**	**76**
Chaetodontidae	Bannerfishes	17	9	26	0	0
	*Prognathodes*	2	9	11	0	0
	*Chaetodon*	52	39	91	0	99
	**Total**	**71**	**57**	**128**	**0**	**71**
Labridae	Labridae 1b	2	10	12	58	92
	Labridae 2b	6	90	96	10	100
	Labridae 2g	7	19	25	48	36
	Other labrids	69	414	484	9	2
	**Total**	**84**	**533**	**617**	**12**	**17**
Pomacentridae	*Lepidozygus*	1	0	1	0	0
	Pomacentridae 1	19	48	67	0	100
	Pomacentridae 2	21	88	109	0	0
	Pomacentridae 3	9	11	20	0	0
	Pomacentridae 4	54	130	184	0	60
	**Total**	**104**	**277**	**381**	**0**	**48**

Percentage of species not represented in the analyses due to missing genera and the percentage of species using Low-quality diet (LQD) in each subclade are also shown.

ClustalX 2.0 [49] was used to generate initial alignments for all gene sequences, which were edited using MacClade 4.0 [50]. After sequence edition, the whole datasets consisted of 1,341 bp for Acanthuroidei; 3,106 bp for Chaetodontidae; 2,736 bp for Labridae; and 4,129 bp for Pomacentridae.

### Phylogenetic analysis

We inferred the phylogenetic relationships within each family through Bayesian Inference (BI) in MrBayes 3.1.2 using the GTR+Γ model [51], [52]. Data was partitioned by gene in Chaetodontidae and Pomacentridae; for Acanthuroidei, both genes were set as a single partition and for Labridae the mitochondrial genes were set as single partition while the nuclear genes were considered as independent partitions. These partitions were chosen using Bayes factors by comparing the following partitioning schemes: 1. all genes apart; 2. mitochondrial genes apart and nuclear genes as a single partition; 3. nuclear genes apart and mitochondrial genes as a single partition; 4. nuclear and mitochondrial genes as single partitions but apart from each other; 5. all genes as a single partition.

To infer each tree using Bayesian Inference, two independent Markov Chain Monte Carlo (MCMC) runs (each with four chains), were implemented simultaneously for 10 million generations, with trees sampled and saved every 1,000 generations (10,000 trees saved per run) using MrBayes [51]. Default settings were used for all other parameters. All trees prior to stationarity and convergence of the runs were discarded. Stationarity was assigned starting at the leveling off of log likelihood scores and convergence was assumed when the value for the standard deviation of split frequencies remained below 0.01. We computed a majority rule consensus tree (50%) and posterior probabilities of clades with the post-burnin trees. All runs were conducted on the CBSU Web Computing Interface (accessed online at http://cbsuapps.tc.cornell.edu/mrbayes.aspx), and results were analyzed with Tracer 1.5 [53].

### Dates estimation and fossil calibration

Divergence times of lineages in all four groups of study were estimated using BEAST 1.5.4 [54]. Rates of lineage splitting were estimated independently from an uncorrelated exponential distribution (UCED) or lognormal distribution (UCLD) given that previous studies tend not to demonstrate autocorrelation of these rates and times of divergence [55]–[57]. We specified a Yule (pure birth) prior to rates of cladogenesis, given that it assumes only a constant speciation rate across the phylogeny. However, given the high effective sample size values for the estimated parameters, the choice of prior is likely to have no influence on the resulting estimates. The partitions described above were assumed to have evolved according to a GTR model with Γ-distributed rate heterogeneity, which was selected based on the Akaike Information Criterion (AIC) values using Modeltest 3.7 [58]. We assigned hard lower bounds and 95% soft upper bounds to the prior distributions of all fossil calibrations points using lognormal distributions. For all calibration points, the hard lower bounds were based on the age of the oldest fossil record of the clade, following the corresponding studies (Table 2).

**Table 2:** Lognormal distribution, priors and references for the calibration points.

Tree	node	hard-lower bound (Ma)	95% soft-upper bound (Ma)	ln(mean)	ln(SD)	References
Acanthuroidei	Acanthuridae	50	65	1.0	1.04	Bellwood, 1996 [14]
Chaetodontidae	Outgroup	50	65	1.0	1.04	Bellwood *et al.*, 2010 [25]
Labridae	Labridae 2b	15	50	1.5	1.25	Bellwood, 1990 [95]; Smith *et al.*, 2008 [96]
	Labridae 1	50	65	1.0	1.04	Bellwood, 1990 [95]; Cowman *et al*., 2009 [72]
	Labridae 2	50	65	1.0	1.04	Bannikov & Sorbini, 1995 [48]; Cowman *et al.*, 2009 [72]
	root	50	65	1.0	0.8	Bellwood, 1990 [95]
Pomacentridae	root	50	65	1.0	0.8	Bellwood, 1990 [95]

Since the vast majority of reported chaetodontid fossils are demonstrably erroneous [59], in the case of Chaetodontidae tree we followed Bellwood *et al.* [39] to assign an 95% soft upper bound of 65 Ma at the outgroup node, which is represented by a range of putative outgroup taxa in the reef fish families Zanclidae, Drepaneidae, Ephippidae, Kyphosidae, Scatophagidae and Pomacanthidae [46]. This age represents the transition of fish faunas at the K/T boundary [46], [60], [61] beyond which there is no fossil record of modern reef fish families. For the same reason, we set the 95% soft upper bound of 65 Ma at the root of Pomacentridae; at the origin of Acanthuridae in the Acanthuroid tree; at the origin of the Labridae 1 clade and at the origin of the Labridae 2 group in the labrid tree (Table 2). The labrid root received the same soft upper bounds but with ln (mean) closer to 65 Ma than 50 Ma. Only in the Labridae 2b node, the 95% soft upper bound was placed at 50 Ma, marking the oldest fossil record of the Labridae [62].

For each of the four groups we used CBSU Web Computing Interface to run three independent analyses of 10 million generations, sampling trees every 500 generations. Resulting log files were examined using Tracer 1.5 [53] to ensure convergence. After excluding the first 10% burnin, tree files were combined using LogCombiner [54]. With the resulting file, we computed a maximum clade credibility tree using TreeAnnotator [54] to display mean node ages and highest posterior density (HPD) intervals at 95% (upper and lower) for each node.

### Character optimization

While it is self-evident that all diets are nutritionally adequate, there are fundamental differences in food composition (i.e. C/N ratio per unit mass) [63], [64], and in the way that nutrition is acquired (i.e. feeding behavior) [15]. We provide a simple binary classification for trophic modes termed low quality and high quality diets: 1) The low quality diet was based on food items that are mainly consumed by herbivores, corallivores and coral mucus feeders, which have significantly more carbohydrate in its composition, high ash content, and relatively low nitrogen per unit mass (i.e. sponge tissues, plant material, detritus, etc.). These items generally present physiological challenges for digestion or relatively inaccessible organic components as a result of structural features and secondary metabolites [22]–[25]. 2) The high quality diet was based on food items that are mainly consumed by piscivores, zooplanktivores and mobile invertebrate feeders, which have relatively high protein/carbohydrate ratio per unit mass [64], [65]. These items also present few physiological challenges for digestion (i.e. fish, crustaceans, annelids, echinoderms, mollusks, etc.), assuming that rigid covering shells and crustacean exoskeletons could be overcome [15].

We have classified all species according to their trophic modes and mapped them onto the BI trees using parsimony, as implemented in Mesquite 2.75 [66]. These traits were also optimized with MP (Maximum Parsimony) analysis to identify the independent evolution of trophic modes.

### Diversification statistics

All diversification statistics were performed in R [67] using APE 3.0-11 [68], GEIGER 1.99-3.1 [69], and LASER 2.4-1 [70] packages. In order to look for general patterns of declining species-level diversification rate, we applied the constant-rates (CR) test [71] to estimate the γ statistic in each of the four BEAST chronograms. The CR test uses a measure of the relative positions of internal nodes within a phylogeny as basis for calculating the value of γ, which is then used to test the null hypothesis of constant per-lineage speciation and extinction rates through time. Within a completely sampled phylogeny the γ values fit a standard normal distribution with mean  =  0, considering a Yule process [71]. Decreases in rates of cladogenesis over time are detected by significantly negative γ values. The incomplete sampling in our analysis will tend to underestimate the number of nodes toward the present, and the CR test may therefore detect a false slowdown in diversification rate. To correct such bias, we performed a Monte Carlo Constant Rates (MCCR) test [71] in which complete phylogenies are simulated under the Yule process by randomly selecting a subsample from the reconstructed topologies and removing them from the analyses. This procedure generates a corrected null distribution allowing the estimation of the significance of negative γ values, which makes the results conservative to extinction. The observed γ values were compared to the 95% confidence interval of a null distribution based on 10,000 topologies simulated for each of the four groups.

We estimated global diversification rate (r**G**) for each of the four groups using the method of Margallon & Sanderson (eqn. 8–11 in [72]). This method calculates the 95% confidence interval of expected species richness for a lineage with origin at time (t) and evolving under a given r**G** and ε, and then compares those estimates with the observed species richness. The estimation considers extinction rates (ε), thus, we estimated r**G** under different values of ε (0.0, 0.5, 0.9 and 0.99). Subclades were defined at the highest monophyletic clades in which species share the same trophic mode. The species richness of such lineages was measured based on valid names listed in the Catalog of Fishes [73] and crown ages were obtained from BEAST chronograms. For lineages with a single species sampled we used stem ages. It is important to use methods with different approaches to correct for possible equivocal interpretation of the results given the limitations of each method. The method of moments' estimator basically considers the relationship between species richness and age. Thus, we used the Relative Cladogenesis (RC) statistic [74] and the method described in [75] to look for significant shifts in net diversification rates. The RC test identifies lineages with significantly faster or slower rates of cladogenesis using a chronogram to calculate the probability that an internal node with n descendent tips get partitioned into two subclades of size *r* and *s*, using a broken-stick distribution as the null expectation [74]. To apply Rabosky's method [75], we first reduced the trees at the level of the subclades of interest by pruning all but one species per subclade from the chronograms. Then, we assigned species richness to each terminal branch as a phenotype vector [70]. This method uses a birth–death estimator based on phylogenetic and taxonomic data to estimate diversification rate and compute the respective likelihood. It contrasts the likelihood of the data under a model with equal diversification rates for all lineages (constant model) to the likelihood under a model where an ancestral diversification rate r1 shifts to a new rate r2 along some branch in the tree (flexible model) [76], [77]. For the flexible model, the tree is sequentially split at each branch and r is optimized to the resulting pair of subtrees. The node resulting in the maximum combined likelihood for the bipartite tree is the ML estimate of the shift point. Analyses were conducted under extinction fractions of ε = 0.0, 0.5, 0.9 and 0.99. To contrast the models we used the likelihood ratio test statistic (χ^2^ distributed with 2 *d.f.*). Our hypothesis predicts that the flexible model would be more likely than the constant model especially when assigning higher net diversification rates at the origin of clades containing low-quality diet feeders. However, it does not necessarily mean that the net diversification rates have increased at that node. Alternatively, the clade could have retained an ancestral but elevated rate, while another clade or clades exhibited a decline in net diversification. To test these scenarios, we used a constrained version of the two-rate model (‘rate-decrease model’), where the highest net diversification rate must occur in the tree bipartition containing the root node, such that no net rate increase is allowed on the path from the root to the clade assigned by the shifting point [78], [79].

## Results

### Phylogenetic Inferences

**Figure 1:**
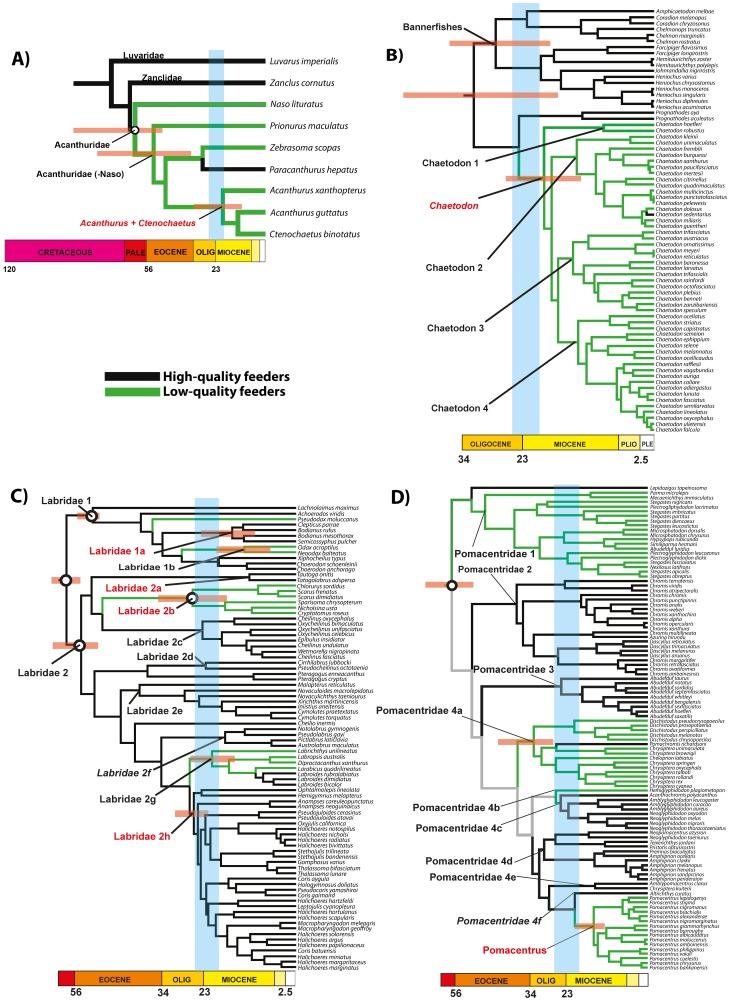
BEAST chronogram showing optimized character under diet quality of: A) Acanthuroidei; B) Chaetodontidae; C) Labridae; D) Pomacentridae. Black branches represent high-quality feeders, green branches represent low-quality feeders and gray branches mean equivocal character. Red names represent subclades that showed significant higher than expected species richness under rG conditioned to different extinction rates. Bars on nodes represent the 95% HPD of subclades mean age. White dots indicate calibration points. The blue bar represents the expansion of IWP reefs in the Oligo-Miocene period among 25–20 Ma [2], [5], [80].

Bayesian inferences produced highly resolved topologies, very close to those found in the original articles. All phylogenies were well supported and presented good resolution for optimization. For all four clades, stationarity and convergence were reached before 10% of the generations. Valid estimations from Mr. Bayes runs were indicated by good effective sample sizes (which shows that the MCMC simulation had an adequate length) in the resulting statistics compiled with Tracer. All resulting phylogenies are shown in Figure 1, and the supporting statistics are presented in Table S1 of the appendix.

### Dates estimation and character optimization

Mean evolutionary rate, Yule process birth rate, coefficients of variation and mean covariance are presented in Table 3 and the resulting chronograms are shown in Figure 1. The coefficients of variation (CV) are used to assess the overall degree of rate heterogeneity across the trees. The resulting posterior densities from the CV were different from zero, indicating significant rate heterogeneity (unclock-like behavior) in all four groups. The mean covariance (MC) was used to assess the degree of autocorrelation of rates across lineages. In all four phylogenies the resulting posterior densities from MC were close to zero, suggesting that branches with fast rates and slow rates are next to each other.

**Table 3:** Mean evolutionary rate (MER), Yule process birth rate (Yule), coefficient of variance (CV) and mean covariance (MC) for each of the ultrametric trees reconstructed using BEAST.

Tree	MER	Yule	CV	MC
	95% HPD	95% HPD	95% HPD	95% HPD
Acanthuroidei	1.24E-3	1.19E-2	0.54	−0.11
	8.2E-4–1.6E-3	6.1E-3–1.8E-2	0.3–0.7	−0.4–0.1
Chaetodontidae	5.51E-3	8.4E-2	0.37	−1.3E-2
	3.9E-3–7.2E-3	5.6E-2–0.1	0.2–0.4	−0.1–0.1
Labridae	3.89E-3	4.7E-2	0.48	7.3E-2
	3.4E-4–4.3E-3	3.6E-2–5.8E-2	0.4–0.5	−0.07–0.2
Pomacentridae	2,66E-3	5.1E-2	0.47	8.7E-2
	2E-4–3.2E-3	3.9E-3–6.5E-2	0.4–0.5	−0.04–0.2

95% HPD  =  high-posterior density interval of 95% for each value estimated.

In the Acanthuroidei, the 95% HPD interval was high for all ages at nodes corresponding to basal relationships. However, the family Acanthuridae had the origin estimated at 61 Ma (95% HPD: 86–50 Ma) (Fig. 1A). Character optimization indicated that the low-quality food habit has evolved once in the suborder at about 52 Ma in the family Acanthuridae (95% HPD: 76–34 Ma) (Fig. 1A). The origin of the most speciose clade, formed by the genera *Acanthurus* + *Ctenochaetus* was 20 Ma (95% HPD: 31–10 Ma).

The origin of chaetodontid outgroup (taxonomically related reef fish families) was estimated at 55 Ma (95% HPD: 65–50 Ma) as we set in our prior. As expected, the family Chaetodontidae seems to be a relatively young family originated at 33 Ma (95% HPD: 45–21 Ma) (Fig. 1B). The relatively low-quality food habit (mainly corallivory) arose at about 20 Ma (95% HPD: 28–13) within the clade *Chaetodon*. The specialization of obligate corallivores appears shortly thereafter at about 15 Ma (95% HPD: 24–12 Ma), in the clade *Chaetodon* 3 (Fig. 1B).

For the labrid tree, three clades used for calibration had age estimates close to the age of the oldest fossil: labrid root  = 58 Ma (95% HPD: 62–55 Ma), Labridae 1 = 51 Ma (95% HPD: 53–50), and Labridae 2 = 54 Ma (95% HPD: 59–50) (Fig. 1C). Character optimization indicated that four lineages have arisen that exploit low-quality diets, all independently and at different times, using different food items, although most of the radiation was during the Miocene (Fig. 1C).

The origin of Pomacentridae estimated at 52 Ma (95% HPD: 61–50 Ma) was very close to the age of the oldest fossil of the family used to set a hard lower bound prior (Fig. 1D). The exploitation of low-quality resources appears at the first split in Pomacentridae 1 + *Lepidozygus*. There are three other clades with low-quality diets: Pomacentridae 4a (stem age  = 31 Ma, 95% HPD: 39–25), Pomacentridae 4b (25 Ma, 95% HPD: 32–19 Ma) and the genus *Pomacentrus* (15 Ma, 95% HPD: 19–11 Ma), from which only *Pomacentrus* seems to have arisen after the Oligo-Miocene transition (Fig. 1D).

### Net diversification rates

**Figure 2:**
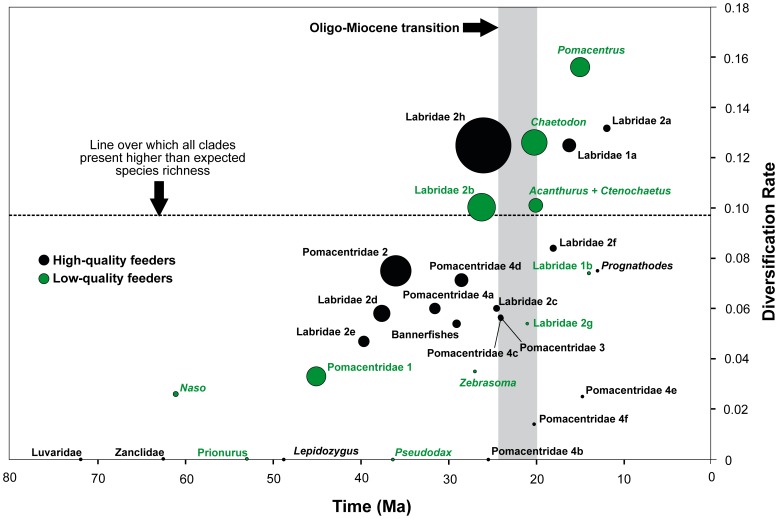
Individual net diversification rate (rG) of subclades *vs*. subclades ages. Black circles represent high-quality feeders, green circles represent low-quality feeders. Dashed line represents the r**G** value over which all clades showed significant higher than expected species richness. Crown ages are according to mean node ages in BEAST analysis. Circle sizes are proportional to the number of species in each clade.

Estimated global net diversification rates (r**G**) decreased with increasing extinction rates, as already noted in the method description. Significantly higher than expected species richness under r**G** conditioned to different ε values are found in Figure 2 (Table S1). It corresponds to: (1) *Acanthurus* + *Ctenochaetus* (Acanthuroidei, Fig. 1A); (2) *Chaetodon* (Chaetodontidae, Fig. 1B); (3) Labridae 1a, (4) Labridae 2a, (5) Labridae 2b and (6) Labridae 2 h (Labridae, Fig. 1C); and (7) *Pomacentrus* (Pomacentridae, Fig. 1D). In the Acanthuroidei, we looked for the species richness formed by the clade Acanthuridae to conduct the test since all species, except *Paracanthurus hepatus*, share the same trophic character in this monophyletic group, and we obtained significant values of *p* only under ε = 0.0. Acanthuridae (-*Naso*) has a mean age of 52 Ma, however, 75% of these species belong to the subclade formed by *Acanthurus* + *Ctenochaetus*, which originated about 20 Ma. Therefore, we believe that all influence that led to higher than expected number of extant species in Acanthuridae (-*Naso*) came exclusively from the effects of the *Acanthurus* + *Ctenochaetus* diversification. We applied the test considering *Acanthurus* + *Ctenochaetus* species richness and we obtained significant values of *p* under even higher extinction rates (ε = 0.0 to ε = 0.99). For Labridae 2 h (Labridae) and *Pomacentrus* (Pomacentridae) we obtained significant values of *p* under ε = 0.0 to ε = 0.9. The remaining clades showed significant *p* values under ε = 0.0 to ε = 0.5 (Table S1). From these seven speciose clades, RC tests indicated higher than expected rates of cladogenesis in *Chaetodon* (Chaetodontidae), *Pomacentrus* (Pomacentridae), Labridae 1a and Labridae 2b (Labridae).

The comparisons between a constant model and a flexible model showed that the flexible model provided the best explanation for elevated net diversification rate at clades *Acanthurus* + *Ctenochaetus* in Acanthuroidei (ε = 0.0 to ε = 0.5), *Chaetodon* in Chaetodontidae (ε = 0.0), Labridae 2 h in Labridae (ε = 0.0 to ε = 0.99) and *Pomacentrus* in Pomacentridae (ε = 0.0 to ε = 0.5) (Table S2).

## Discussion

### Low-quality diets and net diversification rates

Patterns of diversification in the Acanthuroidei, Chaetodontidae and Pomacentridae are all concordant with the hypothesis of density-dependent diversification. Clades that shifted towards a new low-quality diet, and originated during the expansion of IWP coral reefs in the Oligo-Miocene period (25–20 Ma) [2], [5], [80] (Fig. 1, blue line), have a higher than expected number of extant species. These clades are: *Acanthurus* + *Ctenochaetus* in the Acanthuroidei (Fig.1A), *Chaetodon* in the Chaetodontidae (Fig. 1B) and *Pomacentrus* in the Pomacentridae (Fig. 1D). With exception of the Acanthuroidei, these shifts in the rate of diversification have been previously reported [5], [38]. However, this is the first study to quantitatively examine the contribution of trophic modes across all three groups.

It is reasonable to attribute the high diversity found in *Chaetodon*, *Pomacentrus* and *Acanthurus* + *Ctenochaetus* to Oligo-Miocene tectonic events and the expansion of reefs in the IWP [2], [5], [80]. This pattern would be expected considering that most species analyzed in this study are from the IWP and that the origin and diversification of most reef fish lineages took place within regions of the Tethys Sea that later became this biogeographic region during this period [81]. However, many lineages with diets based on high-quality food resources with origins and subsequent radiations close to the Oligo-Miocene boundary did not produce significantly more speciose clades (see Fig. 2 and *Prognathodes* in Chaetodontidae, Fig. 1B; Pomacentridae 3, Pomacentridae 4c, Pomacentridae 4e and Pomacentridae 4f in Pomacentridae, Fig. 1D). Cowman & Bellwood [5] reported that families with a higher proportion of coral-dwelling species had higher rates of diversification following a possible cryptic extinction event. It is now clear that reef systems have been major drivers in reef fish evolution most likely by providing broader range of niches to be exploited [4], [56]. Our results suggest that exploitation of under-used and abundant ‘low-quality’ food resources may have played a significant role in this context. Ecological or trophic opportunism may have been a key factor contributing to high diversification rates on specific reef fish groups.

Our results show remarkable parallels to recent studies of fossil surgeonfishes (Acanthuridae) and rabbitfishes (Siganidae) [82] which report a marked change in the trophic status of these fishes in the Oligocene to early Miocene, with the suggestion that the early formation of modern coral reef fish faunas was associated with the exploitation of underutilized EAM (epilithic algal matrix) resources on coral reef flats. The movement onto reef flats was part of a general increase in the exploitation of low-quality trophic resources at that time.

Within the Labridae, the low-quality feeders of the subclade Labridae 2b, which encompasses *Scarus* and *Chlorurus*, showed higher than expected net speciation rates. Several explanations for the relatively high rates of diversification in this subclade have been proposed, including the presence of an intermandibular joint [83], which may be related to the efficient exploitation of a low-quality diet. Relatively high rates of diversification may also reflect vicariance and social factors which may also enhance speciation within the subclade [84]. However, two other subclades of Labridae with low-quality diets that originated during, or after, the Oligo-Miocene transition (Labridae 1b, Labridae 2g), did not presented higher than expected net diversification rates. These results suggest that patterns of diversification within the Labridae may also be influenced by variables such as limited or constrained morphological diversity in Labridae 2g [85], or being a temperate lineage (Labridae 1b) [86]. Moreover, three high-quality diet subclades (Labridae 1a, Labridae 2a and Labridae 2h), presented higher than expected net diversification rates (Fig. 1C, Fig. 2). Clearly, while some labrid groups may have been influenced by a shift to low quality diets, other factors are important and have driven diversification in a range of subclades exploiting a range of trophic modes. These factors may also include biogeographic phenomena such as the expansion of reefs in the Indo-Australian Archipelago or an associated shift in the distribution of reef fishes into the Central Indo-Pacific [2], [5], [84], [87].

It is hard to determine whether low-quality diets are taxonomically more diverse than high-quality diets, given that fishes often exploit complex resources rather than specific food items [88], [89]. The key to exploitation of these resources, however, appears to be the possession of the appropriate morphological and physiological modifications to be able to harvest and process low-quality (and often particulate) resources. Indeed, recent fossil evidence has highlighted the Oligocene/Miocene as a key time for the acquisition of morphological features that enable the exploitation of low-quality diets in fishes [90], and the invasion of new areas with extensive availability of low-quality material on coral reefs [82]. These shifts to low-quality diets are associated with the appearance of jaws for scraping in parrotfishes [4], [83], [91], long-toothed morphologies for selectively harvesting soft tissues or particulates in chaetodontids, acanthurids and blennies [90], and a change on head or body shape to permit exploitation of exposed locations in surgeonfishes [82]. These shifts are likely to be supported by a range of physiological changes in the gut to accommodate the new challenges presented by low-quality diets [26], [29], [92]. Overall, our results and previous work all point to the Oligocene to early Miocene as a key period in the evolution of reef fishes, marked by a transition to low-quality diets. This trophic shift also appears to have been associated with relatively high rates of diversification.

### Competitive interactions and the origin of low-energy diets

There is a central conceptual difference between the present study and that of Harmelin-Vivien [16] in terms of the evolutionary role of decreased competition due to the use of low-quality food resources. For Harmelin-Vivien [16], the rise of low-quality diets is an adaptive response to high levels of competition. In our study, high levels of competition are not necessarily related to the rise of lineages exploiting low-quality diets, but it may be related to the higher net diversification rates in these lineages.

Evolutionary delayed origins of low-energy diets seems to be a widespread pattern within marine communities and has already been described for major metazoan clades such as Crustacea, Mollusca and Echinodermata in different phylogenetic scales [93]. These other clades might have also contributed to high levels of competition on Oligocene/Miocene coral reefs, given that some of these lineages expanded during this period [2] and that they have the ability to compete with fishes for food resources. Within fishes delayed origins of low-quality diets has previously been reported in the Labridae [87] and Chaetodontidae [38]. We now expand this to other major fish groups, showing that the nature and timing of these trophic shifts varies considerably, and that these shifts did not just occur in the Oligocene-Miocene.

The patterns seen in the Acanthuroidei, Chaetodontidae and Labridae are all consistent with the suggestion that the use of low-quality food resources is a derived condition which has evolved independently at least once in each clade [94]. However, the origin of this condition does not appear to be temporally congruent among clades, as suggested by Harmelin-Vivien [16]. In the Acanthuroidei, the origin of low-quality diets in the Acanthuridae coincides with the origin of many other reef fish families (approximately 65–50 Ma), and occurs much earlier than the main period of diversification in reef fishes in the Miocene [5] when most modern reef fish genera arose (Fig. 1A). These results are robust even considering the missing species in the analyses, because all recognized genera are included and the percentage of species using low-quality diets in each subclade are proportional to those used in the reconstructions (Table 1). The Pomacentridae displays the same temporal pattern, with low-quality diet apparently represented at the root of the tree, with the possibility of several independent convergences to a high or low-quality diet (Fig. 1D) [35], [36]. It should be noted that in Pomacentridae the different food habits (herbivory, omnivory and zooplanktivory) are likely to be found in all lineages, varying only in the relative contribution of zooplankton and algae to the total ingested biomass.

By contrast, chaetodontid (and to some extent labrid) evolution seems to agree quite well with Harmelin-Vivien's [16] predictions, where the use of relatively low-quality food resources would be a derived condition that arose when reef communities experienced high species richness, suggested by the origin and radiation of *Chaetodon* during the Miocene (Fig. 1B). The reconstruction of ancestral trophic character states suggests that the first butterflyfish may have had the ability to use low-quality food items but could count on a little contribution of high-quality food resources in their diet. Only later did some lineages become more restricted, feeding exclusively on corals, with at least four independent origins of this feeding mode [38]. A similar pattern is seen in the labrids with two independent herbivorous lineages [87].

In the Chaetodontidae, as in Pomacentridae, feeding behaviour appears to be similar among lineages. All *Chaetodon* spp. bite the substratum to ingest food, and are primarily distinguished from each other by the surface they bite. However, the dietary differentiation among chaetodontid lineages seems to transition evolutionarily from high to low-quality diets (possibly due to competitive interactions [16]), while in pomacentrids, low-quality diets are found among early diverging, intermediate and young lineages.

The observations in the present study support recent findings that suggest that reef fishes have experienced two major phases of trophic development in the Cenozoic: an early phase in the Paleogene and a later phase in the Oligocene-Miocene [82], [87], [90]. Interestingly, the exploitation of low-quality foods was involved in both phases with the Acanthuridae and Pomacentridae exploiting these resources in the Paleogene and the Chaetodontidae and Labridae transitioning primarily in the Neogene. Clearly, the exploitation of low-quality resources is a shift that has been a repeated feature in reef fishes over the last 60 million years.

## Conclusions

Our study suggests that the diversification of reef fish lineages may be shaped by ecological opportunity. We present a new perspective in which the emergence of low-quality feeding modes may underpin diversification in a broad range of reef fish families. Our study reinforces the idea of density-dependent diversification influencing the radiation of clades. Moreover, we suggest that trophic shifts have not necessarily induced increasing net rates of diversification in speciose clades, but that they may have reduced declines. Species-rich clades may, therefore, have maintained diversification over time while other clades decreased when niches became saturated. Overall, our study emphasizes the importance of trophic opportunism and low-quality diets in the evolution of reef fishes.

## Supporting Information

Table S1 **Subclades with higher than expected number of extant species indicated by global diversification rate.** Subclades with higher than expected number of extant species indicated by the test of Magallon & Sanderson [63]. **rG**  =  estimated global diversification rate under different values of extinction rate (**ε**). ***p***<0.05 indicates significant speciose subclades. **NS**  =  not significant. Subclade labeled in bold  =  subclades with species using low-quality diets.
(DOCX)

Table S2 **Likelihoods and Akaike Information Criterion for constant and flexible models of diversification under different extinction rates.** Likelihoods (LH) and Akaike Information Criterion (**AIC**) for constant and flexible models of diversification under different extinction rates (**ε**). **r** is the constant diversification rate estimated across all lineages. p<0.05 indicates significant higher likelihood estimated for the flexible model, in which a diversification rate **r1** shift to **r2** at the subclade indicated. **NS** indicates that the flexible model was not significant assuming shifts in any of the subclades. Rate decreasing scenario was not significant in any case, except in Pomacentridae over ε = 0.5, which was not considered due the equivocal phylogenetic position of *Lepidozygus*.
(DOCX)
