# Supplementary material for: Diet and Diversification in the Evolution of Coral Reef Fishes
Source: PLoS One. 2014 Jul 16;9(7):e102094. doi: 10.1371/journal.pone.0102094 (PMC4100817; doi:10.1371/journal.pone.0102094)
Supplement: Table S1 — Subclades with higher than expected number of extant species indicated by global diversification rate. Subclades with higher than expected number of extant species indicated by the test of Magallon & Sanderson [63]. rG = estimated global diversification rate under different values of extinction rate (ε). p<0.05 indicates significant speciose subclades. NS = not significant. Subclade labeled in bold = subclades with species using low-quality diets. (DOCX) [file pone.0102094.s001.docx]

**SUPPORTING INFORMATION**

**Table S1.**

| **Tree** | **Subclade** | **Nº species** | **Age (Ma)** | ***p*** | | | |
| --- | --- | --- | --- | --- | --- | --- | --- |
|  |  |  |  | **ε=0** | **ε=0.5** | **ε=0.9** | **ε=0.99** |
| Acanthuroidei |  |  |  | rG=0.035 | rG=0.032 | rG=0.021 | rG=0.006 |
|  | Luvaridae | 1 | 71.9 | NS | NS | NS | NS |
|  | Zanclidae | 1 | 62.5 | NS | NS | NS | NS |
|  | *Naso* | 19 | 61.1 | NS | NS | NS | NS |
|  | **Acanthuridae (-*Naso*)** | 62 | 52.5 | 0.3E-3 | 0.6E-2 | NS | NS |
|  | ***Acanthurus* + *Ctenochaetus*** | 47 | 20 | 0.9E-12 | 0.2E-7 | 0.7E-3 | 0.4E-1 |
| Chaetodontidae |  |  |  | rG=0.124 | rG=0.116 | rG=0.076 | rG=0.024 |
|  | Bannerfishes | 26 | 29 | NS | NS | NS | NS |
|  | *Prognathodes* | 11 | 13 | NS | NS | NS | NS |
|  | ***Chaetodon*** | 91 | 20.2 | 0.5E-2 | 0.3E-1 | NS | NS |
|  | **Chaetodon 1** | 2 | 9.2 | NS | NS | NS | NS |
|  | **Chaetodon 2** | 37 | 14.2 | 0.1E-1 | 0.4E-1 | NS | NS |
|  | **Chaetodon 3** | 21 | 15 | NS | NS | NS | NS |
|  | **Chaetodon 4** | 31 | 14 | NS | NS | NS | NS |
| Labridae |  |  |  | rG=0.098 | rG=0.093 | rG=0.070 | rG=0.033 |
|  | ***Pseudodax*** | 1 | 36.3 | NS | NS | NS | NS |
|  | Labridae 1a | 48 | 16.2 | 0.3E-3 | 0.8E-2 | NS | NS |
|  | **Labridae 1b** | 12 | 14 | NS | NS | NS | NS |
|  | Labridae 2a | 26 | 11.9 | 0.1E-2 | 0.2E-1 | NS | NS |
|  | **Labridae 2b** | 96 | 26.2 | 0.5E-2 | 0.3E-1 | NS | NS |
|  | Labridae 2c | 23 | 24.5 | NS | NS | NS | NS |
|  | Labridae 2d | 60 | 37.6 | NS | NS | NS | NS |
|  | Labridae 2e | 39 | 39.6 | NS | NS | NS | NS |
|  | Labridae 2f | 24 | 18 | NS | NS | NS | NS |
|  | Labridae 2g | 9 | 21 | NS | NS | NS | NS |
|  | *Labroides* | 5 | 8.4 | NS | NS | NS | NS |
|  | **Labridae 2g**  **(-*Labroides*)** | 14 | 21 | NS | NS | NS | NS |
|  | Labridae 2h | 200 | 26 | 0.2E-7 | 0.4E-3 | 0.3E-1 | NS |
| Pomacentridae |  |  |  | rG=0.097 | rG=0.092 | rG=0.066 | rG=0.029 |
|  | *Lepidozygus* | 1 | 48.7 | NS | NS | NS | NS |
|  | **Pomacentridae 1** | 67 | 45 | NS | NS | NS | NS |
|  | Pomacentridae 2 | 109 | 36 | NS | NS | NS | NS |
|  | Pomacentridae 3 | 20 | 24 | NS | NS | NS | NS |
|  | **Pomacentridae 4a** | 40 | 31.5 | NS | NS | NS | NS |
|  | **Pomacentridae 4b** | 1 | 25.4 | NS | NS | NS | NS |
|  | Pomacentridae 4c | 19 | 24 | NS | NS | NS | NS |
|  | Pomacentridae 4d | 48 | 28.5 | NS | NS | NS | NS |
|  | Pomacentridae 4e | 4 | 14.7 | NS | NS | NS | NS |
|  | Pomacentridae 4f | 2 | 20.2 | NS | NS | NS | NS |
|  | ***Pomacentrus*** | 70 | 15 | 0.3E-8 | 0.1E-3 | 0.3E-1 | NS |
