# Supplementary material for: Diet and Diversification in the Evolution of Coral Reef Fishes
Source: PLoS One. 2014 Jul 16;9(7):e102094. doi: 10.1371/journal.pone.0102094 (PMC4100817; doi:10.1371/journal.pone.0102094)
Supplement: Table S2 — Likelihoods and Akaike Information Criterion for constant and flexible models of diversification under different extinction rates. Likelihoods (LH) and Akaike Information Criterion (AIC) for constant and flexible models of diversification under different extinction rates (ε). r is the constant diversification rate estimated across all lineages. p<0.05 indicates significant higher likelihood estimated for the flexible model, in which a diversification rate r1 shift to r2 at the subclade indicated. NS indicates that the flexible model was not significant assuming shifts in any of the subclades. Rate decreasing scenario was not significant in any case, except in Pomacentridae over ε = 0.5, which was not considered due the equivocal phylogenetic position of Lepidozygus. (DOCX) [file pone.0102094.s002.docx]

**SUPPORTING INFORMATION**

**Table S2.**

| **Tree** | **ε** | **Constant model** | | | **Flexible model** | | | | | |
| --- | --- | --- | --- | --- | --- | --- | --- | --- | --- | --- |
|  |  | **LH** | **AIC** | **r** | **Subclade** | **LH** | **AIC** | **r1** | **r2** | ***p*** |
| Acanthuroidei | **0.0** | -63 | 129 | 0.043 | *Acanthurus* + *Ctenochaetus* | -58 | 123 | 0.033 | 0.082 | 0.4E-2 |
|  | **0.5** | -63 | 128 | 0.033 | *Acanthurus* + *Ctenochaetus* | -58 | 123 | 0.025 | 0.068 | 0.1E-1 |
|  | **0.9** | -63 | 128 | 0.015 | - | - | - | - | - | NS |
|  | **0.99** | -64 | 130 | 0.002 | - | - | - | - | - | NS |
|  |  |  |  |  |  |  |  |  |  |  |
| Chaetodontidae | **0.0** | -36 | 74 | 0.150 | *Chaetodon* | -33 | 72 | 0.097 | 0.191 | 0.4E-1 |
|  | **0.5** | -35 | 73 | 0.119 | - | - | - | - | - | NS |
|  | **0.9** | -36 | 74 | 0.057 | - | - | - | - | - | NS |
|  | **0.99** | -37 | 76 | 0.009 | - | - | - | - | - | NS |
|  |  |  |  |  |  |  |  |  |  |  |
| Labridae | **0.0** | -116 | 235 | 0.106 | Labridae 2h | -104 | 214 | 0.084 | 0.192 | 0.5E-7 |
|  | **0.5** | -115 | 232 | 0.086 | Labridae 2h | -105 | 216 | 0.068 | 0.171 | 0.6E-6 |
|  | **0.9** | -114 | 231 | 0.047 | Labridae 2h | -108 | 223 | 0.035 | 0.113 | 0.2E-2 |
|  | **0.99** | -119 | 240 | 0.010 | Labridae 2h | -114 | 234 | 0.006 | 0.040 | 0.6E-2 |
|  |  |  |  |  |  |  |  |  |  |  |
| Pomacentridae | **0.0** | -79 | 160 | 0.109 | *Pomacentrus* | -72 | 151 | 0.093 | 0.211 | 0.1E-2 |
|  |  |  |  |  | *Lepidozygus* | -73 | 153 | 0.113 | 0.001 | 0.4E-2 |
|  | **0.5** | -78 | 158 | 0.088 | *Lepidozygus* | -73 | 152 | 0.092 | 0.001 | 0.6E-2 |
|  |  |  |  |  | *Pomacentrus* | -73 | 152 | 0.075 | 0.177 | 0.8E-2 |
|  | **0.9** | -78 | 158 | 0.047 | *Lepidozygus* | -74 | 154 | 0.050 | 0.001 | 0.2E-1 |
|  | **0.99** | -81 | 164 | 0.009 | - | - | - | - | - | NS |
